# Proteomic analysis of granulomas from cattle and pigs naturally infected with *Mycobacterium tuberculosis* complex by MALDI imaging

**DOI:** 10.3389/fimmu.2024.1369278

**Published:** 2024-07-03

**Authors:** Fernanda Larenas-Muñoz, José María Sánchez-Carvajal, Inés Ruedas-Torres, Carmen Álvarez-Delgado, Karola Fristiková, Francisco José Pallarés, Librado Carrasco, Eduardo Chicano-Gálvez, Irene Magdalena Rodríguez-Gómez, Jaime Gómez-Laguna

**Affiliations:** ^1^ Department of Anatomy and Comparative Pathology and Toxicology, Pathology and Immunology Group (UCO-PIG), Unidad de Investigación Competitiva (UIC) Zoonosis y Enfermedades Emergentes ENZOEM, University of Córdoba, Córdoba, Spain; ^2^ Pathology Group, United Kingdom Health Security Agency (UKHSA), Salisbury, United Kingdom; ^3^ Instituto Maimónides de Investigaciones Biomédicas (IMIBIC) Mass Spectrometry and Molecular Imaging Unit (IMSMI), Maimónides Biomedical Research Institute of Córdoba, Reina Sofia University Hospital, University of Córdoba, Córdoba, Spain

**Keywords:** MALDI-MSI, animal tuberculosis, cattle, pig, *Mycobacterium tuberculosis* complex

## Abstract

Matrix-assisted laser desorption/ionization mass spectrometry imaging (MALDI-MSI) has recently gained prominence for its ability to provide molecular and spatial information in tissue sections. This technology has the potential to uncover novel insights into proteins and other molecules in biological and immunological pathways activated along diseases with a complex host–pathogen interaction, such as animal tuberculosis. Thus, the present study conducted a data analysis of protein signature in granulomas of cattle and pigs naturally infected with the *Mycobacterium tuberculosis* complex (MTC), identifying biological and immunological signaling pathways activated throughout the disease. Lymph nodes from four pigs and four cattle, positive for the MTC by bacteriological culture and/or real-time PCR, were processed for histopathological examination and MALDI-MSI. Protein identities were assigned using the MaTisse database, and protein–protein interaction networks were visualized using the STRING database. Gene Ontology (GO) analysis was carried out to determine biological and immunological signaling pathways in which these proteins could participate together with Kyoto Encyclopedia of Genes and Genomes (KEGG) analysis. Distinct proteomic profiles between cattle and pig granulomas were displayed. Noteworthy, the GO analysis revealed also common pathways among both species, such as “Complement activation, alternative pathway” and “Tricarboxylic acid cycle”, which highlight pathways that are conserved among different species infected by the MTC. In addition, species-specific terms were identified in the current study, such as “Natural killer cell degranulation” in cattle or those related to platelet and neutrophil recruitment and activation in pigs. Overall, this study provides insights into the immunopathogenesis of tuberculosis in cattle and pigs, opening new areas of research and highlighting the importance, among others, of the complement activation pathway and the regulation of natural killer cell- and neutrophil-mediated immunity in this disease.

## 1 Introduction

Animal tuberculosis (TB) is a chronic disease caused by bacteria belonging to the *Mycobacterium tuberculosis* complex (MTC), which has a major global impact due to its importance in public health and the large associated economic losses (1–4). Currently, the World Organization for Animal Health (WOAH) considers all mammalian species susceptible to TB, with the ability of any member of the MTC to induce a highly similar disease (5). The tuberculous granuloma serves as a pathological hallmark during the development of the disease (6, 7). According to the multi-host dimension of TB (5, 8, 9), different animal models have been used to unravel the host immune response (10–15). However, the interaction between host and mycobacterium is complex, with numerous gaps in our understanding of the pathogenesis of this disease. Therefore, the use of new methodological approaches is encouraged to improve the understanding of the mechanisms involved in the development of TB.

Matrix-assisted laser desorption/ionization mass spectrometry imaging (MALDI-MSI) has emerged as a novel methodology for this purpose, being capable of simultaneously detecting biomolecules by their molecular weight, such as proteins, lipids, metabolites, and other biological macromolecules, together with their spatial tissue distribution (16–18). In this sense, MALDI-MSI has been mostly applied to clinical research, tumor classification, biomarker identification, molecular histology, and metabolism, among other areas of interest (19, 20). In the context of TB, this tool has been already employed in the search for *M. tuberculosis* virulence factors (21) as well as in biomarker and drug resistance studies in humans (15).

The multi-host character of animal TB (5, 8, 9) highlights the likely activation of common pathways in the different animal species infected by mycobacteria belonging to the MTC (14, 22–24). Nonetheless, structural peculiarities have also been reported in concrete species (7, 10, 11, 13, 14, 25); therefore, species-specific pathways are equally expected to be activated along this disease.

The flexibility of MALDI-MSI has allowed the detection of analytes in different tissues and structures (17, 18), such as the identification of the cytokine environment in granulomas from humans and rabbits (14). Thus, the confirmation of specific molecules involved in the activation of specific pathways will enlighten the pathogenesis of the disease, as well as the recognition of biomarkers of interest for its diagnosis and control. Therefore, the present study aims to compare the protein signature in granulomas from lymph nodes of cattle and pigs naturally infected by the MTC, identifying the potential participation of these proteins in biological and immunological pathways activated along the disease, together with those differentially expressed in both species.

## 2 Experimental procedures

Ethical review and approval were not required for this animal study since no purposed killing of animals was addressed.

### 2.1 Animals and tissue samples

For this study, samples from 14- to 16-month-old, male and female, Limousin bovine and Iberian pig lymph nodes (LNs) from the Spanish national program for surveillance and monitoring of bovine TB and from free-range pig carcasses completely condemned at the slaughterhouse due to the presence of generalized tuberculosis-like lesions (TBLs) were used (25, 26). Samples, which corresponded to four tracheobronchial LNs from cattle and four mandibular LNs from pigs, were fixed in 10% neutral buffered formalin for histopathological and histomolecular studies (MALDI-MSI). All included animals resulted positive for the MTC by bacteriological culture and/or real-time PCR (qPCR) from porcine (25) and bovine (26) samples according to previous results. The tuberculous granuloma was considered the experimental unit.

### 2.2 Histopathology

Four-micrometer sections were stained with H&E for histopathological examination. Additionally, another set of sections was stained by the Ziehl–Neelsen (ZN) technique for the identification of acid-fast bacillus (AFB). H&E-stained sections were used to identify microscopic TBLs, and the ZN technique was considered positive by detecting at least one AFB in at least one high-power field magnification (HPF, 100×). Positive samples were classified as paucibacillary (1–10 AFB) or pluribacillary (≥11 AFB) as previously reported (27).

### 2.3 MALDI-MSI technique

#### 2.3.1 Sample preparation for MALDI-MSI

Three-micrometer sections of each sample were mounted onto an indium tin oxide slide (576352, Sigma-Aldrich, Darmstadt, Germany) previously coated with poly-l-lysine (P1274–25mg, Sigma-Aldrich). Following this, a standard deparaffinization/rehydration protocol was carried out as follows: two washes of 10 min in xylene, one wash of 5 min in absolute ethanol, 96% and 70%, followed by two additional washes of 5 min each in 10 mM ammonium bicarbonate (NH_4_HCO_3_). Subsequently, antigen retrieval was performed by heating the rehydrated tissues with 100 mM Tris, pH 9, at 98°C for 30 min. Samples were allowed to cool at room temperature until reaching a minimum temperature of 50°C and then subjected to three additional 3-min washing steps with 10 mM NH_4_HCO_3_ prior to on-tissue digestion.

#### 2.3.2 On-tissue digestion and matrix deposition

On-tissue digestion was carried out by spraying four layers of trypsin (V5111, Promega, Madison, WI, USA) at a concentration of 0.1 µg/µL in 25 mM NH_4_HCO_3_ and 10% trifluoroethanol (Sigma-Aldrich), maintaining a constant flow of 10 µL/min using a SunCollect sprayer (SunChrom, Friedrichsdorf, Germany). Afterward, samples were incubated overnight at 37°C within a saturated humid chamber. Following the completion of the on-tissue digestion, the slides were vacuum-dried for 30 min before matrix deposition. A matrix solution containing 7 mg/mL with 60% acetonitrile (Fisher Chemical, Waltham, MA, USA) and 0.2% trifluoroacetic acid α-cyano-4-hydroxycinnamic acid (HCCA; Sigma-Aldrich) was employed to cover the tissues. Additional internal calibrants, namely, bradykinin F1–7, angiotensin II, and glu-fibrinopeptide (Sigma-Aldrich), were added to the matrix solution.

The final matrix solution was applied using the SunCollect sprayer in eight layers, as follows: the first layer at 10 µL/min, the second layer at 20 µL/min, and the third to eighth layers at 30 µL/min, all at a z-axis equal to 27.05. Upon completion, the slides underwent vacuum drying for an additional 30 min.

#### 2.3.3 Sample processing for MALDI-MSI

Imaging measures were carried out in positive ionization mode using an ultrafleXtreme mass spectrometer (Bruker Daltonics, Bremen, Germany).

The *m*/*z* range for all samples was set from 700 to 2,500 *m*/*z*. A laser intensity global attenuator was fixed to 20%, and the number of shots per pixel was fixed to 600. A digitizer was fixed to 2.50GS/s, real-time smoothing was set to medium, and baseline offset adjustment was fixed to 3.2% or 4.2 mV. Voltage parameters were adjusted for each sample to ensure a resolution full width at half maximum (FWHM) >15,000 at the glu-fibrinopeptide reference mass peak. For mass spectrometer calibration purposes, four reference peaks previously mixed with the HCCA matrix were used. Those peaks were 757.3998 [M+H]^+^ (bradykinin F1–7), 842.508 [M+H]^+^ (trypsin autolysis peak), 1,046.5420 [M+H]^+^ (angiotensin II), and 1,570.6770 [M+H]^+^ (glu-fibrinopeptide). Calibration was conducted by fixing a maximum peak tolerance error of 50 ppm, and quadratic mode was used for peak adjustment.

All datasets were acquired using the flexImaging software (version Bruker, Germany) with a lateral resolution of 100 μm for all samples, enough to allow a correct analysis distinguishing different histological features. Tissue samples were analyzed in a random order to prevent any possible bias due to factors such as variation in mass spectrometer sensitivity or matrix influence.

#### 2.3.4 Data analysis

For MALDI-MSI generation, SCILS^®^ analysis software (Bruker, Germany) was first used to export the entire acquired tissue areas to imzml file format to share them in the ProteomeXchange public repository (28). Later, the same dataset was used to perform a “virtual microdissection” of regions of interest (ROIs) containing granulomas by doing bisecting k-means segmentation of the entire scanned tissue area. After this microdissection, each ROI was saved in imzml file format. Thereafter, the Cardinal R package (v3.0.1) was used (29) to analyze the granuloma dataset. Data files were normalized by total ion current (TIC), and all spectra underwent baseline subtraction to remove noise, resampling via peak-picking to lower data dimensionality, and smoothing to remove tissue and measurement artifacts. Then, a mean spectrum for each granuloma was extracted for statistical analysis. The Cardinal package was used to generate molecular images based on the Viridis linear color scale, identifying the more intense positive signal intensity of the peptide in question in intense yellow color.

After data generation, *m*/*z* features obtained from granulomas in cattle and pigs, meaning common *m*/*z* found in granulomas from both species and *m*/*z* from each species, were evaluated. For this purpose, the online Venn Diagram platform of the University of Ghent, Belgium, was used (https://bioinformatics.psb.ugent.be/webtools/Venn/). Afterward, the reference MaTisse database (30) was used to assign the identity of putative proteins to the molecular weight of each *m*/*z* with a margin of ±0.025 *m*/*z* or 30 ppm. The gene coding for each selected protein was then identified on the UniProt platform (https://www.uniprot.org/).

Statistical analysis was performed using MetaboAnalyst 4.0 software (https://www.metaboanalyst.ca/) using a one-way analysis of variance (ANOVA) test. Datasets were structured according to the developer’s instructions. Datasets were previously normalized including sample median normalization, logarithmic transformation, and auto-scaling. Sparse partial least squares discriminant analysis (sPLS-DA) with a cross-validation (CV) error rate of 2.6% was used to illustrate the separation between the groups. Additionally, hierarchical clustering and heatmap that allowed visualization of the MALDI-MSI analysis were also performed. All software used in our analysis has open access from their corresponding author repositories.

### 2.4 Construction of protein–protein interaction network

The interaction between identified proteins from cattle and pig granuloma was visualized by protein–protein interaction (PPI) networks using the STRING database (Search Tool for the Retrieval of Interacting Genes) (31) through Cytoscape software (32). STRING protein is an online tool that integrates information from multiple protein–protein association databases and provides interaction predictions (31). Only consistent interactions were considered for a cut-off point ≤0.4.

### 2.5 GO analysis

For the visualization of protein molecular interaction networks, the Cytoscape software (version 3.91) was used (32). Functional analysis of proteins involved in granulomas from cattle and pigs was performed using the plugins ClueGO (version 2.5.9) and CluePedia (version 1.5.9) (33, 34) for detailed information on pathways. Plugins for ClueGO describing biological processes (BPs), immune system processes (ISPs), and Kyoto Encyclopedia of Genes and Genomes (KEGG) pathways integrated with Gene Ontology (GO) were used together as an enrichment step. Significantly represented pathways were visualized into ClueGO functionally grouped networks. Regarding this, following instructions (35), the *Homo sapiens* organism was selected for having the most extensive mapped genes, and different levels of specificity were used. Specific pathways with GO level tree interval 7–15 and GO term/pathway selection with a minimum of 3 proteins/term and at least 15% of coverage from the total associated proteins were selected. To carry out GO analysis from common proteins between both species, GO level tree interval 3–8 and GO term/pathway selection with a minimum of three proteins/term and at least 3% of coverage from the total associated proteins were selected. Differentially expressed proteins (DEPs) were obtained from the dataset, showing the DEPs whose pathways were statistically significant (*p* < 0.05). For analysis, a log2 fold-change (FC) was performed using the data obtained to evaluate differentially expressed *m*/*z* after MALDI-MSI, considering a false discovery rate (FDR) <0.001 and a log2 FC ≥ 2 to determine those *m*/*z* that were expressed in a differential way between both species with statistical significance. The differentially expressed *m*/*z* were visualized in a volcano plot, which was performed using GraphPad Prism 9.0 software (GraphPad Prism software 9.0, Inc., San Diego, CA, USA).

For GO, a kappa score ≤0.4 was used to define term–term interactions (edges) and functional groups based on shared proteins between the terms on the network. *p* < 0.05 was calculated using a right-sided hypergeometric test with Bonferroni step-down correction for multiple testing.

### 2.6 Validation of MALDI-MSI results by immunohistochemistry

Immunohistochemistry (IHC) against the protein complement 3 (C3) in bovine, human leukocyte antigen–DR (HLA-DR) in pig, and matrix metalloproteinase 9 (MMP9) in both species was used to validate MALDI-MSI results. Four-micrometer tissue sections were deparaffinized with xylene and rehydrated with graded alcohols followed by blocking endogenous peroxidase activity with 3% hydrogen peroxide in methanol for 30 min. For antigen retrieval, the samples were heated in citrate buffer (10 mM, 0.05% Tween 20, pH 6.0) for C3, HLA-DR (10 mM, pH 3.2), and MMP9 (10 mM, pH 6.0). After this, sections were incubated with 2% bovine serum albumin (BSA) blocking solution for 30 min. Primary antibodies [anti-C3c rabbit polyclonal antibody (Abcam, Cambridge, UK) with a 1:100 dilution in 2% BSA, anti-human HLA-DR mouse monoclonal antibody, clone TAL.1B5 (Dako, Copenhagen, Denmark) with a 1:25 dilution in 2% BSA, and anti-MMP9 rabbit polyclonal antibody (Thermo Fisher Scientific, Hsinchu, Taiwan) with a 1:100 dilution in 2% BSA] were applied and incubated overnight at 4°C. For C3 immunolabeling, the HRP kit (Immunohistochemical detection from kit Cell Signalling,Technology Inc., MA, USA) was applied and incubated 30 min after washes with 1× Tris-buffered saline with 0.1% Tween 20 (TBST). Visualization was performed using DAB (Dako). For HLA-DR and MMP9 immunolabeling, biotinylated goat anti-mouse (Dako) and goat anti-rabbit IgG (Vector Laboratories, Burlingame, CA, USA) secondary antibodies were diluted 1:200 in 2% BSA, respectively, and applied for 30 min after washing in phosphate-buffered saline (PBS). Then, avidin–biotin–peroxidase complex (ABC Vector Elite, Vector Laboratories) was applied and incubated for 1 hour at room temperature. Immunolabeling was visualized by application of the NovaRED™ substrate kit (Vector Laboratories). The slides were counterstained with Harris hematoxylin, dehydrated, and mounted using EUKITT^®^ mounting medium (Sigma-Aldrich).

### 2.7 Experimental design and statistical rationale

The samples were taken from a retrospective study, considering a total of four routinely slaughtered cattle and four pigs. After veterinary inspection, LNs were collected and processed for histopathology and microbiological culture for MTC detection. The final samples were used as described in the experimental setup and consisted of a retrospective targeted sampling, with samples being positive for the MTC according to the gold standard technique for TB diagnosis (microbiological culture/qPCR) and with tuberculous granuloma at histopathology. The tuberculous granuloma was considered as the experimental unit, then WinEpi 2.0 (Faculty of Veterinary, University of Zaragoza, Spain; http://www.winepi.net/uk/index.htm) was used with a confidence level of 95% and a 12.00% margin of error in order to determine the sample size, obtaining a minimum sample size 67 granulomas for each species. In the case of MALDI-MSI analysis, all the slides were performed in triplicate.

For MALDI-MSI and GO terms, statistical data are shown in the different sections of this study. The differences were considered statistically significant when *p* ≤ 0.05.

## 3 Results

### 3.1 Histopathology and identification of the matrix proteome from granulomas in cattle and pigs

A total of 220 and 78 granulomas in the LNs from cattle and pigs, respectively, naturally infected with the MTC were identified. The cellular composition of granulomas was similar in both species, but a higher presence of neutrophils in the center of the granuloma and infiltrating the connective tissue of the surrounding capsule was observed in porcine granulomas. All samples from both species showed AFB positivity, displaying a paucibacillary pattern in both bovine and porcine granulomas, apart from one cattle in which a pluribacillary pattern was also observed.

**Figure 1:**
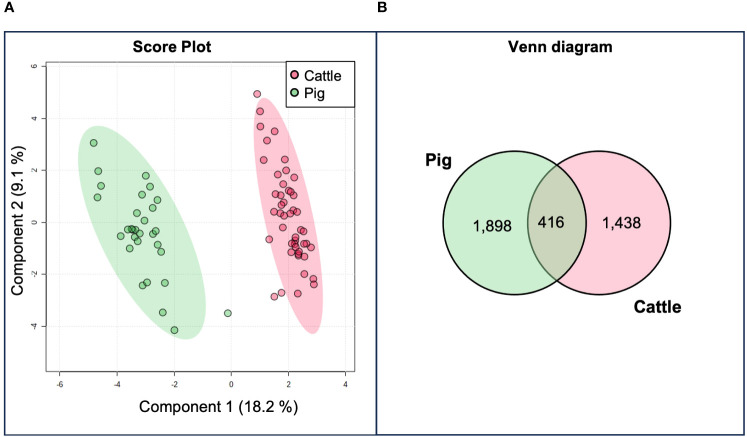
**(A)** Sparse partial least squares discriminant analysis (sPLS-DA). Scattered red and green dots correspond to bovine and porcine granulomas, respectively, distributed according to their protein signature. A clear separation between bovine and pig granulomas is evident. The explained variances for each component are shown in brackets. **(B)** Venn diagram showing the distribution of *m*/*z* features present in cattle and pigs, obtained from matrix-assisted laser desorption/ionization mass spectrometry imaging (MALDI-MSI) analysis and identifying species-specific *m*/*z* as well as those common *m*/*z* features among both species.

After MALDI-MSI analysis, a total of 4,168 *m*/*z* features were obtained from cattle and pig granulomas, showing two clearly separated groups according to the clustering and distribution of the features within each species when evaluated by sPLS-DA (**Figure 1A**). These *m*/*z* were distributed as follows: 1,438 *m*/*z* exclusively in cattle granulomas, 1,898 *m*/*z* only in porcine granulomas, and 416 *m*/*z* shared between both species (832 *m*/*z* in total) (**Figure 1B**). After comparison of the molecular weight from each *m*/*z* value with the reference MaTisse database, a total of 433 potential proteins out of the 1,438 specific *m*/*z* were identified in cattle, 732 potential proteins out of the 1,898 specific *m*/*z* were identified in porcine, and 115 potential proteins out of the 416 *m*/*z* were identified as shared between both species.

### 3.2 Construction of PPI network and functional annotation analysis in cattle

**Figure 2:**
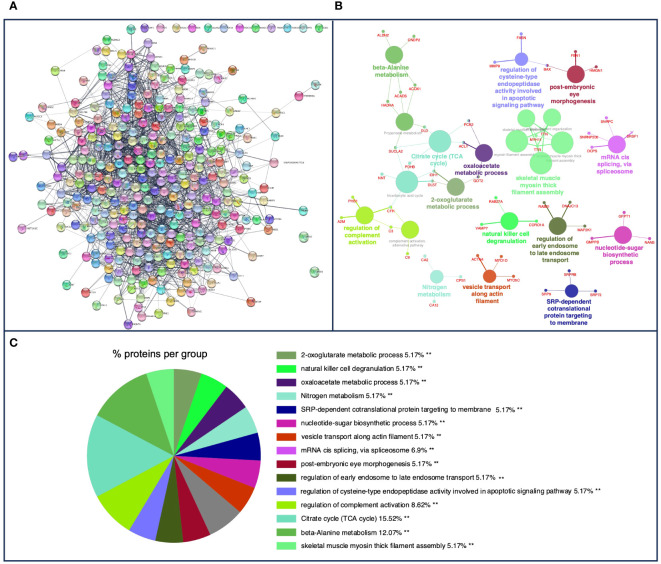
**(A)** Protein–protein interaction (PPI) network of 323 overlapped nodes and 1,971 edges from bovine-specific *m*/*z* features obtained using the STRING database and visualized using Cytoscape. **(B)** Functional network from cattle-specific *m*/*z* features was visualized using Cytoscape with ClueGo and CluePedia, incorporating biological processes (BPs), immune system processes (ISPs), and Kyoto Encyclopedia of Genes and Genomes (KEGG) for enrichment of the pathways. Terms are displayed as nodes (colored circles), with node size being directly proportional to *p* ≤ 0.05. Terms are linked by edges (lines) based on their kappa score. Only statistically significant terms have been considered. **(C)** Sector diagram representing the proportion of proteins associated with the top functional groups expressed in a pie chart according to Gene Ontology (GO) terms specific to each group. A value of *p* ≤ 0.05 shows significantly enriched GO terms. **p ≤ 0.01.

**Figure 2A** shows the PPI network in cattle, where the interaction of 323 nodes and 1,971 edges was obtained using the STRING database. Afterward, a functional enrichment analysis based on the GO database was performed to explore BPs and ISPs together with KEGG analysis. Twenty-two significantly enriched GO terms with a *p* < 0.05 were identified (**Figure 2B**). These terms were clustered into 15 different groups according to their expression level and were represented according to the percentage of proteins per group (**Figure 2C**). The results of the GO analysis showed that most of the *m*/*z* in cattle were mainly involved in the citrate cycle [tricarboxylic acid (TCA) cycle] (KEGG:00020), tricarboxylic acid cycle (GO:0006099), regulation of complement activation (GO:0030449), natural killer cell degranulation (GO:0043320), nitrogen metabolism (KEGG:00910), regulation of cysteine-type endopeptidase activity involved in apoptotic signaling pathway (GO:2001267), vesicle transport along actin filament (GO:0030050), complement activation, alternative pathway (GO:0006957), and regulation of early endosome to late endosome transport (GO:2000641), among others. **Table 1** shows these terms associated with their corresponding putative proteins identified in cattle. All terms identified in bovine granulomas together with the proteins included within each term can be consulted in **Supplementary Table 1**.

**Table 1:** Summary list with the terms from Gene Ontology (GO) biological processes (BPs), immune system processes (ISPs), and Kyoto Encyclopedia of Genes and Genomes (KEGG) and their specific *m*/*z* identified in cattle.

GO term	No. proteins	% Associated proteins	Associated proteins found
Citrate cycle (TCA cycle)	7	23.33	[ACLY, DLD, DLST, IDH1, PCK2, PDHB, SUCLA2]
Tricarboxylic acid cycle	6	17.65	[CFH, DLST, IDH1, NNT, PDHB, SUCLA2]
Regulation of complement activation	4	18.18	[A2M, C3, CFH, PHB1]
Natural killer cell degranulation	3	23.08	[CORO1A, RAB27A, VAMP7]
Nitrogen metabolism	3	17.65	[CA12, CA2, CPS1]
Regulation of cysteine-type endopeptidase activity involved in apoptotic signaling pathway	3	16.67	[BAX, FASN, MMP9]
Vesicle transport along actin filament	3	15.79	[ACTN4, MYO1D, MYO5C]
Complement activation, alternative pathway	3	15.00	[C3, C9, CFH]
Regulation of early endosome to late endosome transport	3	15.00	[DNAJC13, MAP2K1, RAB21]

For the complete list see **Supplementary Table 1**.

No., number.

### 3.3 Construction of PPI network and functional annotation analysis in pigs

**Figure 3:**
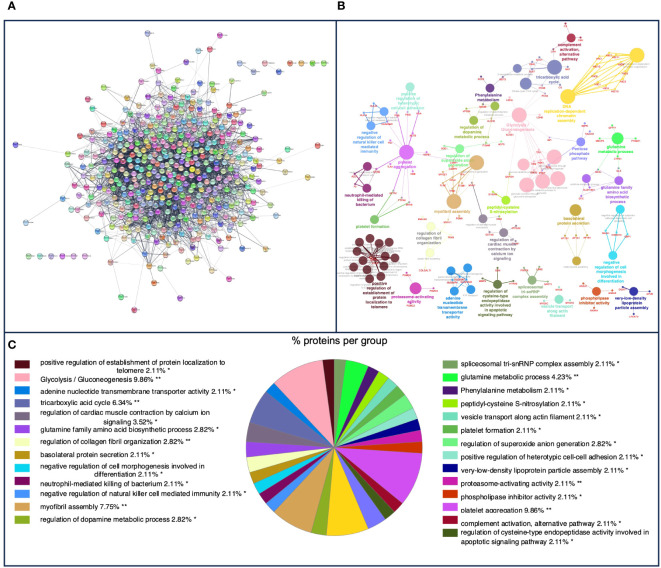
**(A)** Protein–protein interaction (PPI) network of 447 overlapped nodes and 3,994 edges from pig-specific *m*/*z* features obtained using the STRING database and visualized using Cytoscape. **(B)** Functional network from pig-specific *m*/*z* features was visualized using Cytoscape with ClueGo and CluePedia, incorporating biological processes (BPs), immune system processes (ISPs), and Kyoto Encyclopedia of Genes and Genomes (KEGG) for enrichment of the pathways. Terms are displayed as nodes (colored circles), with node size being directly proportional to *p* ≤ 0.05. Terms are linked by edges (lines) based on their kappa score. Only statistically significant terms have been considered. **(C)** Sector diagram representing the proportion of proteins associated with the top functional groups expressed in a pie chart according to Gene Ontology (GO) terms specific to each group. A value of *p* ≤ 0.05 shows significantly enriched GO terms. *p ≤ 0.05; **p ≤ 0.01.

In pig granulomas, a higher number of *m*/*z* was observed in the PPI network, which showed the interaction of 447 nodes and 3,994 edges (**Figure 3A**). Afterward, according to the GO functional enrichment (BP and ISP categories) and KEGG analyses, most of the *m*/*z* were significantly enriched within 61 different terms (**Figure 3B**), which merged into 29 different groups (**Figure 3C**). The main terms, in which these proteins were involved, were glycolysis/gluconeogenesis (KEGG:00010), platelet aggregation (GO:0070527), tricarboxylic acid cycle (GO:0006099), neutrophil-mediated killing of bacterium (GO:0070944), regulation of cysteine-type endopeptidase activity involved in apoptotic signaling pathway (GO:2001267), negative regulation of natural killer cell mediated cytotoxicity (GO: 0045953), negative regulation of natural killer cell mediated immunity (GO:0002716), platelet formation (GO:0030220), and complement activation, alternative pathway (GO:0006957). **Table 2** shows these terms associated with the putative proteins identified in each term. An extensive description of all terms identified in pig granulomas with the proteins included for each term is detailed in **Supplementary Table 2**.

**Table 2:** Summary list with terms from Gene Ontology (GO) biological processes (BPs), immune system processes (ISPs), and Kyoto Encyclopedia of Genes and Genomes (KEGG) and their specific *m*/*z* identified in pigs.

GO term	No. proteins	% Associated proteins	Associated proteins found	
Glycolysis/gluconeogenesis	14	20.90	[ACSS1, ALDH2, ENO3, GAPDH, GPI, HK2, LDHA, LDHB, PCK2, PDHB, PFKL, PFKP, PGK1, TPI1]
Platelet aggregation	14	18.42	[ACTB, ACTN1, CEACAM1, CSRP1, CTSG, FGA, FGB, FGG, FN1, HBB, HSPB1, MYH9, PTPN6, TLN1]
Tricarboxylic acid cycle	7	20.59	[ACO1, CFH, CS, DLST, IDH2, NNT, PDHB]
Neutrophil-mediated killing of bacterium	3	25	[AZU1, CTSG, F2]
Regulation of cysteine-type endopeptidase activity involved in apoptotic signaling pathway	3	16.67	[GSN, HTRA2, MMP9]
Negative regulation of natural killer cell mediated cytotoxicity	3	15,79	[CEACAM1, HLA-A, HLA-B]
Negative regulation of natural killer cell mediated immunity	3	15	[CEACAM1, HLA-A, HLA-B]
Platelet formation	3	15	[ACTN1, MYH9, PTPN6]
Complement activation, alternative pathway	3	15	[C8G, C9, CFH]

For the complete list, see **Supplementary Table 2**.

No., number.

### 3.4 Construction of PPI network and functional annotation analysis from common *m*/*z* identified in both cattle and pigs

**Figure 4:**
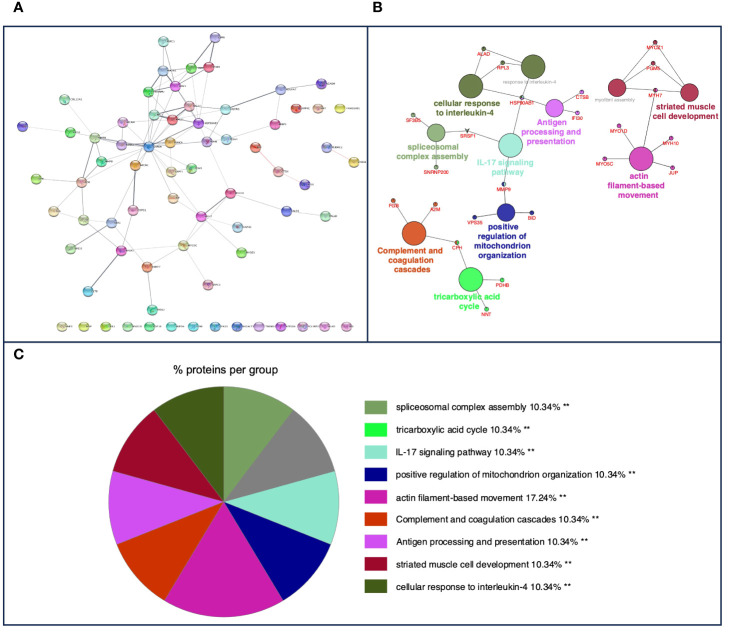
**(A)** Protein–protein interaction (PPI) network of 71 overlapped nodes and 93 edges from common *m*/*z* features among bovine and porcine obtained using the STRING database and visualized using Cytoscape. **(B)** Functional network from cattle and pig common *m*/*z* features was visualized using Cytoscape with ClueGo and CluePedia, incorporating biological processes (BP), immune system processes (ISPs), and Kyoto Encyclopedia of Genes and Genomes (KEGG) for enrichment of the pathways. Terms are displayed by nodes (colored circles), with node size being directly proportional to *p*-value ≤0.05. Terms are linked by edges (lines) based on their kappa score. Only statistically significant terms have been considered. **(C)** Sector diagram representing the proportion of proteins associated with the top functional groups from cattle and pig common *m*/*z* expressed in a pie chart according to Gene Ontology (GO) terms specific to each group. A value of *p* ≤ 0.05 shows significantly enriched GO terms. **p ≤ 0.01.

A total of 416 *m*/*z* were shared in granulomas from both species, in which a PPI network with a total of 71 nodes and 93 edges was obtained (**Figure 4A**). In the functional analysis of the proteins, after GO functional enrichment (BP and ISP categories) and KEGG analyses, all *m*/*z* were significantly enriched in a total of 11 different terms (**Figure 4B**), which merged into nine different groups (**Figure 4C**), highlighting the response to interleukin-4 (GO:0070670), antigen processing and presentation (KEGG:04612), complement and coagulation cascades (KEGG:04610), IL-17 signaling pathway (KEGG:04657), and tricarboxylic acid cycle (GO:0006099). The details of the 11 terms identified in this part of the study together with their putative proteins are shown in **Table 3**.

**Table 3:** List with terms from Gene Ontology (GO) biological processes (BPs), immune system processes (ISPs), and Kyoto Encyclopedia of Genes and Genomes (KEGG) and their specific *m*/*z*, which were shared by both cattle and pigs.

GO term	No. proteins	% Associated proteins	Associated proteins found
Actin filament-based movement	5	3.65	[JUP, MYH10, MYH7, MYO1D, MYO5C]
Response to interleukin-4	3	76.92	[ALAD, HSP90AB1, RPL3]
Striated muscle cell development	3	42.25	[MYH7, MYOZ1, PGM5]
Spliceosomal complex assembly	3	39.47	[SF3B5, SNRNP200, SRSF1]
Antigen processing and presentation	3	38.46	[CTSB, HSP90AB1, IFI30]
Positive regulation of mitochondrion organization	3	38.46	[BID, MMP9, VPS35]
Complement and coagulation cascades	3	35.29	[A2M, CFH, FGB]
IL-17 signaling pathway	3	31.91	[HSP90AB1, MMP9, SRSF1]
Tricarboxylic acid cycle	3	8.82	[CFH, NNT, PDHB]
Cellular response to interleukin-4	3	8.33	[ALAD, HSP90AB1, RPL3]
Myofibril assembly	3	4.29	[MYH7, MYOZ1, PGM5]

No., number.

### 3.5 Differentially expressed proteins in cattle and pig granulomas

**Figure 5:**
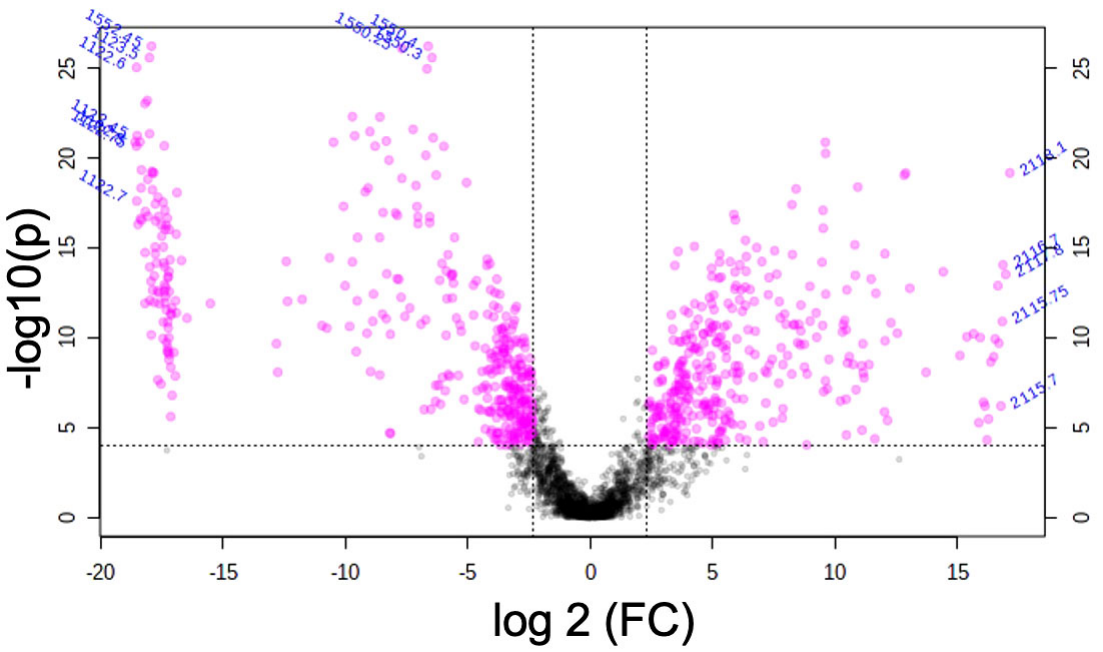
Volcano plot representation of the *m*/*z* features obtained from bovine and porcine granulomas by matrix-assisted laser desorption/ionization mass spectrometry imaging (MALDI-MSI). Magenta dots represent differentially expressed *m*/*z* in both species, while black dots correspond to non-differentially expressed *m*/*z* [false discovery rate (*FDR*) <0.0001 and log2 FC ≥ 2].

DEPs in both species were obtained considering a statistical significance of *FDR* < 0.0001 and a log2 FC ≥ 2. Thus, 436 differentially expressed *m*/*z* were observed in the study, with 187 DEPs in bovine granulomas and 249 DEPs in porcine granulomas (**Figure 5**). From them, 45 putative proteins in cattle and 57 putative proteins in pigs were identified using the reference MaTisse database.

**Figure 6:**
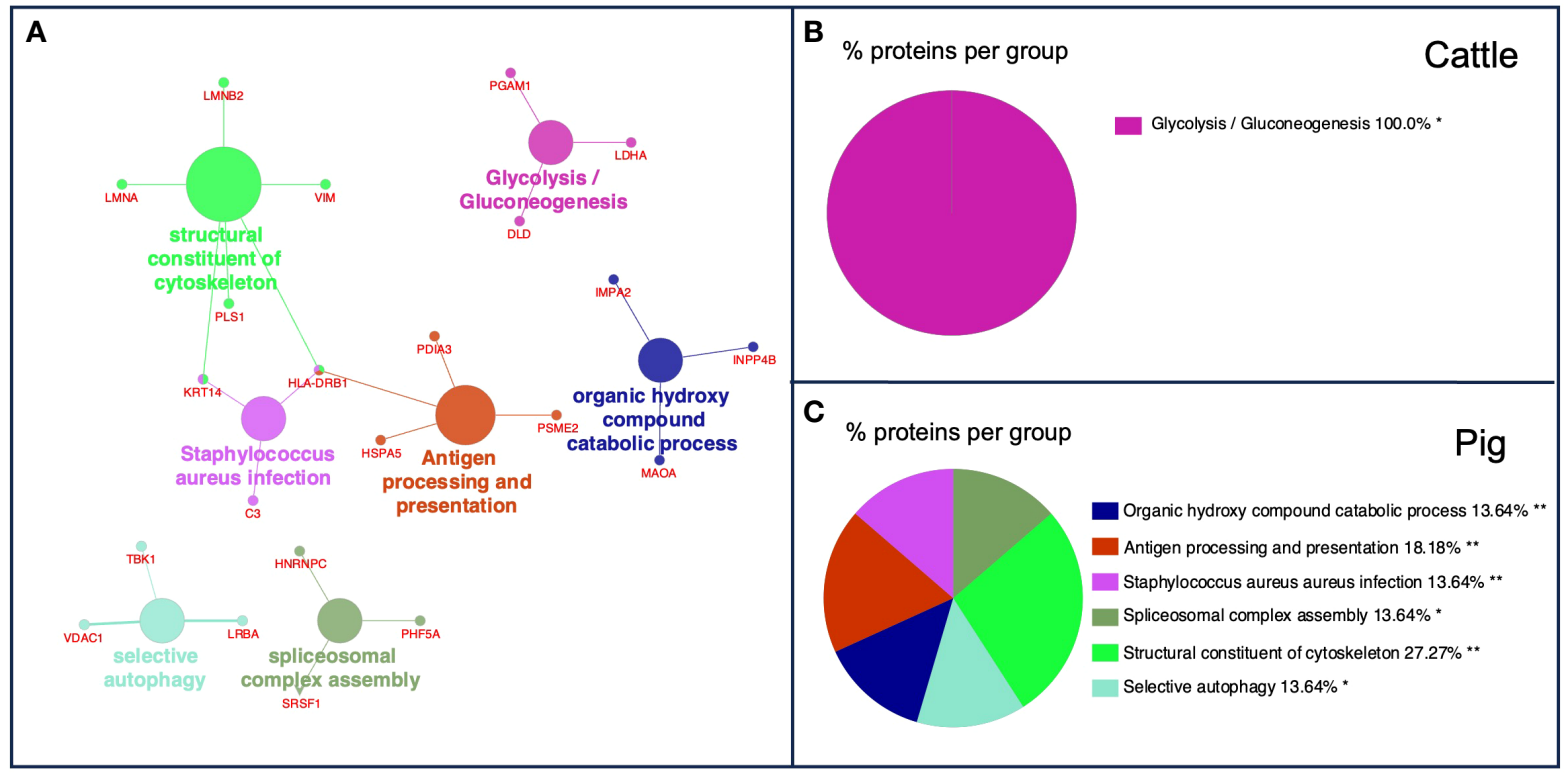
**(A)** Functional network from cattle and pig differentially expressed *m*/*z* features was visualized using Cytoscape with ClueGo and CluePedia, incorporating biological processes (BPs), immune system processes (ISPs), and Kyoto Encyclopedia of Genes and Genomes (KEGG) for enrichment of the pathways. Terms are displayed by nodes (colored circles), with node size being directly proportional to *p*-value ≤0.05. Terms are linked by edges (lines) based on their kappa score. Only statistically significant terms have been considered. **(B, C)** Sector diagram representing the proportion of proteins associated with the top functional groups from cattle **(B)** and pig **(C)** differentially expressed *m*/*z* displayed in a pie chart according to Gene Ontology (GO) terms specific to each group. *p* ≤ 0.05 shows significantly enriched GO terms. *p < 0.05; **p ≤ 0.01.

After GO functional enrichment (BP and ISP categories) and KEGG analyses, the DEPs identified in cattle and pig granulomas were significantly enriched in a total of seven different terms (**Figure 6A**). In cattle, all the *m*/*z* were associated with one single representative term, glycolysis/gluconeogenesis (KEGG:00010) (**Figure 6B**), whereas DEPs from pig granulomas were included within six representative terms, highlighting antigen processing and presentation (KEGG:04612), selective autophagy (GO:0061912), structural constituent of cytoskeleton (GO:0005200), spliceosomal complex assembly (GO:0000245), and organic hydroxy compound catabolic process (GO:1901616) (**Figure 6C**, **Table 4**).

**Table 4:** List of terms of Gene Ontology (GO) biological processes (BPs), immune system processes (ISPs), and Kyoto Encyclopedia of Genes and Genomes (KEGG) and their specific *m*/*z* from differentially expressed proteins in cattle and pigs.

GO term	No. proteins	% Associated proteins	Associated proteins found
Cattle			
Glycolysis/gluconeogenesis	3	44.78	[DLD, LDHA, PGAM1]
Pig			
Antigen processing and presentation	4	51.28	[HLA-DRB1, HSPA5, PDIA3, PSME2]
Structural constituent of cytoskeleton	4	35.71	[HLA-DRB1, KRT14, LMNA, LMNB2]
Spliceosomal complex assembly	3	39.47	[HNRNPC, PHF5A, SRSF1]
Organic hydroxy compound catabolic process	3	35.29	[IMPA2, INPP4B, MAOA]
Selective autophagy	3	34.48	[LRBA, TBK1, VDAC1]
*Staphylococcus aureus* infection	3	31.25	[C3, HLA-DRB1, KRT14]

No., number.

### 3.6 Confirmation of MALDI-MSI results by IHC

**Figure 7:**
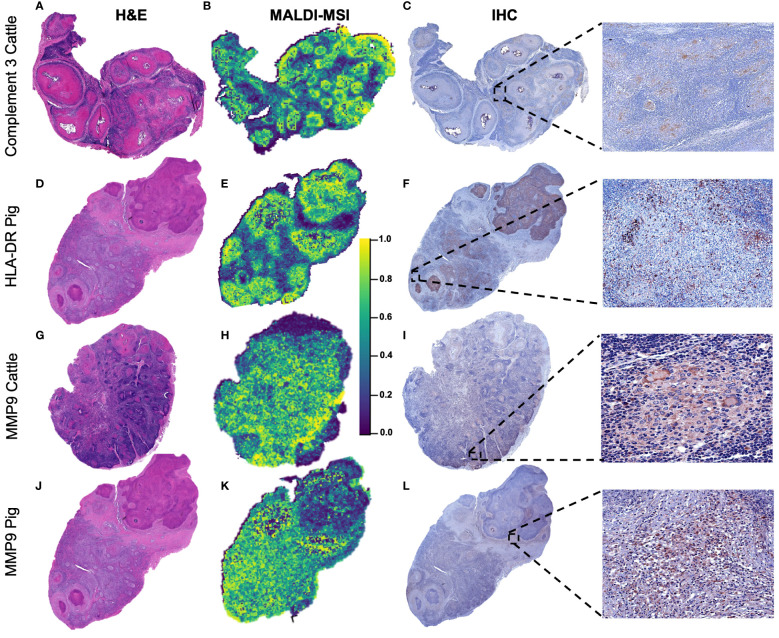
Spatial distribution according to selected *m*/*z* features in bovine and porcine granulomas and validation by immunohistochemistry. **(A, D, G, J)** Sub-gross H&E-stained sections from a lymph node with multiple granulomas belonging to cattle or pigs, as specified. **(B, E, H, K)** Distribution pattern of C3 (*m*/*z* = 2,217.25) in a bovine lymph node (LN), HLA-DR (*m*/*z* = 1,963.90) in a porcine LN, and metalloproteinase 9 (MMP9) (*m*/*z* = 959.40) in bovine and porcine LNs. C3 and HLA-DR were homogeneously distributed throughout granulomas, whereas MMP9 was absent in the necrotic center of granulomas. **(C, F, I, L)** Immunolabeling against the three selected molecules in the different granulomas in each species to confirm matrix-assisted laser desorption/ionization mass spectrometry imaging (MALDI-MSI) results.

**Figure 7** shows the H&E image together with the spatial distribution of C3, HLA-DR, and MMP9 by MALDI-MSI together with the protein expression of each molecule by IHC in different representative animals from the corresponding species. C3 (*m*/*z* = 2,217.25) was mainly expressed in the periphery of the granulomas from cattle (**Figure 7B**); in accordance with the MALDI-MSI image, C3 immunolabeling was found in the cytoplasm of epithelioid macrophages and multinucleated giant cells of granulomas with lack of expression at the necrotic core (**Figure 7C**). In the case of HLA-DR (*m*/*z* = 1,963.95) (**Figures 7D–F**), the spatial distribution and pattern of expression was like C3 but in pig granulomas (**Figure 7E**). Most immunolabeled cells corresponded to epithelioid macrophages organizing the granuloma and located close to the necrotic core (**Figure 7F**). For MMP9 (*m*/*z* = 959.404) spatial distribution in bovine (**Figures 7G–I**) and porcine (**Figures 7J–L**) granulomas, epithelioid macrophages, multinucleated giant cells, and neutrophils and fibroblasts from the periphery of granulomas were immunolabeled in both species (**Figures 7I, L**).

## 4 Discussion

New high-throughput sequencing techniques are emerging as tools of interest to decipher host–pathogen interactions within the context of major diseases, such as TB. In this sense, the combination of sequencing strategies together with tissue morphology, like MALDI-MSI, can provide valuable information to deepen our understanding of the pathogenesis of diseases and how specific lesions are developed. In the current study, a proteomic analysis in tuberculous granulomas from cattle and pig LNs was performed to gain more information on the pathways involved in the regulation of the host response to the disease in each species.

Although only four LNs for each species were considered, this resulted in 220 and 78 granulomas for cattle and pigs, respectively. From them, a similar number of *m*/*z* was identified in both species (1,438 *m*/*z* from cattle granulomas *vs*. 1,898 *m*/*z* from pig granulomas), which allowed us to perform a robust comparison among cattle and porcine. From the beginning of the study, after sPLS-DA, a clear separation between bovine and porcine granulomas according to the expressed *m*/*z* in each species was observed, which already pointed to a different protein signature in granulomas according to the species.

Although specific *m*/*z* were identified in each species, some GO-enriched terms also matched in both. This was the case of the term “Complement activation, alternative pathway” (**Tables 1**, **2**) represented in both species by the proteins complement 9 (C9) and complement factor H (CFH) as well as by the proteins C3 specifically in bovine and complement C8 gamma chain (C8G) in porcine. Opsonophagocytic activity mediated by C3 or C3b has been reported to be enhanced in TB caused by either *M. tuberculosis* (36) or *Mycobacterium bovis* in wild boar, suggesting a role in bacterial clearance, which may be due to the production of IL-1β and other cytokines by innate immune cells, stimulating the production of C3 (37). The expression of different molecules of the alternative pathway of complement activation in both species points to the activation of common pathways with species-specific peculiarities. Another term represented in both species was “Regulation of cysteine-type endopeptidase activity involved in apoptotic signaling pathway”, including MMP9 in both species, but also the proteins FASN and BAX in bovine and GSN and HTRA2 in porcine. Among them, matrix metalloproteinases (MMPs) are significant mediators of the inflammatory response and tissue destruction in TB (38), with MMP9 being specifically involved in macrophage recruitment and tissue remodeling, which allows the formation of the granuloma (39–41). Furthermore, MMP9 has been highlighted as a specific biomarker for the diagnosis of TB in cattle (42, 43), which could be also the case for porcine according to our results. The same applies to several pathways related to the regulation of the metabolism, such as the GO terms “Tricarboxylic acid cycle” (TCA) and “Glycolysis/gluconeogenesis”, which were commonly represented in both species, but again being represented by different proteins. Our findings support a central role for these terms in the metabolism, model proposed by Xu et al. (2022) for *M. tuberculosis*, since amino acids, such as arginine, are synthesized from TCA using carbon metabolites from glycolysis as an energy source, which support the significant role of these pathways in TB in bovine and porcine. Interestingly, and in conjunction with these previous GO terms, “Nitrogen metabolism” was another term activated in cattle, involving proteins such as carbonic hydrogenase-like proteins (CA12 and CA2) and carbamoyl phosphate synthase-1 (CPS-1), which has been shown to play a relevant role in mycobacterial nutrition and survival (44).

Regarding specific terms activated in cattle, the GO term “Natural killer cell degranulation” included the proteins VAMP27, CORO1A, and RAB27A. Degranulation of natural killer (NK) cells involves the release of their granules, containing cytotoxic proteins, such as perforin and granzyme, which trigger the target cells to undergo apoptosis (45). In TB, the release of these granules has been found to control mycobacterial growth, being essential for inhibiting mycobacterial replication (46). On the contrary, “Negative regulation of natural killer cell mediated immunity” and “Negative regulation of natural killer cell mediated cytotoxicity” (HLA-A, HLA-B, and CEACAM1) were activated among porcine GO terms, referring to a set of mechanisms that downregulate or inhibit the activity of NK cells through downregulation of activating receptors on the surface of NK cells or upregulation of inhibitory receptors (45). These pathways may act as a double-edged sword. On the one hand, they contribute to maintaining immune homeostasis and avoiding autoimmunity, thereby preventing excessive inflammation and tissue damage (47). On the other hand, these pathways can be exploited by pathogens to evade the immune system and establish chronic infections, such as TB. Further studies need to be conducted to explore the role of NK cells during TB in swine.

Other specific GO terms identified in cattle included “Vesicle transport along actin filament” (ACTN4, MYO1D, and MYO5C) and “Regulation of early endosome to late endosome transport” (DNAJC13, MAP2K1, and RAB21), built through proteins that participate in endosomal protein and vesicular trafficking. Actin filaments and endosomes are important in TB infection, as they play a critical role in the intracellular trafficking and survival of mycobacteria within host cells (48–50). In addition, an alteration of actin filaments could impede the process of nitric oxide synthase induction and inhibit enzyme activity in activated macrophages, thus increasing the survival of mycobacteria within macrophages (49). Therefore, we could deduce that the integrity of actin filaments for the transport of early endosomes plays an important role in the destruction of mycobacteria (50).

Noteworthy, specific GO terms were activated only in porcine, such as “Platelet formation” and “Platelet aggregation”. Platelets are capable of initiating and accelerating a rapid innate immune response against *M. tuberculosis*, activating monocytes and leading to the expression of activation markers, such as MMPs, as well as phagocytosis and tissue injury (51), in which thrombocytosis and increased severity of the disease may be critical (52). Furthermore, “Neutrophil-mediated killing of bacterium” was also represented in pig granulomas in our study, which is consistent with the histological features observed in this species. In TB, neutrophils are fundamental mediators of the innate immune response, as they provide protection trying to kill mycobacteria through the release of their granules, notably, the primary (azurophilic) granules, which contain myeloperoxidase (MPO), cathepsin G, elastase, proteinase 3, and defensin, responsible for pathogen clearance (53, 54). Interestingly, the proteins azurocidin 1 (AZU1) and cathepsin G (CTSG) were identified in our study within this GO term. Previous studies in cattle have shown active phagocytosis of *M. bovis* by neutrophils; however, neutrophils failed to kill mycobacteria, proposing a potential role for autophagy (55). Remarkably, in the context of mycobacterial infection, neutrophils have been reported to act as a “Trojan horse” with infected neutrophils serving as vehicles, transporting mycobacterial organisms to distant sites, thereby causing systemic dissemination of the bacteria (24, 53, 55).

The GO terms represented by common *m*/*z* in both species (**Table 3**) included “Response to interleukin-4” and “IL-17 signaling pathway”; while the first has an anti-inflammatory function, the second has proinflammatory activity (56). IL-4 has been highlighted as a marker of virulence and protective immune response during TB in cattle, being associated with animals with mild lesions compared to those with severe lesions (57). This supports the idea that IL-4 acts to attenuate the adverse effect of exacerbated interferon gamma (IFN-γ)-driven inflammation in host tissues (58), which surprisingly was not represented in the pathways detected through the analysis. Similar results have been seen in Bacillus Calmette–Guérin (BCG)-immunized wild boars, suggesting implications for the development of vaccines against TB in wild boars and other wildlife species (59). Furthermore, it has been shown that cattle with macroscopic lesions developed higher IL-17 expression in later stages of the disease compared to animals without lesions (58), indicating that IL-17 is an important proinflammatory cytokine in the immune response against mycobacterial infection with predictive prognostic value in TB in cattle (23, 58).

The GO term “Antigen processing and presentation” contained within its proteins cathepsin B (CTSB) and gamma-interferon-inducible lysosomal thiol reductase (IFI30). Cathepsins are proteolytic enzymes with a key role in lysosome formation, contributing to pathogen clearance either directly by pathogen killing or indirectly by participating in antigen presentation. In this sense, a decrease in cathepsin B has been associated with an increased survival of intracellular mycobacteria (60). Furthermore, cathepsin B has been reported to be pivotal in granuloma immunopathology during TB (61).

In addition, DEPs among both species were analyzed. Due to the low number of DEPs identified using the MaTisse database, only the term “Glycolysis/gluconeogenesis” was recognized among GO terms in cattle. Interestingly, gluconeogenesis is known as essential for mycobacteria survival in the host (62), with a central role of phosphoenolpyruvate carboxykinase for *M. tuberculosis* infections and survival (63). The differential expression of this term in granulomas from cattle indicates a key role of this metabolic pathway in bovine for the metabolism of mycobacteria from the MTC. In the case of pigs, among the GO terms represented from DEPs, “Antigen processing and presentation” and “Structural constituent of cytoskeleton” were found, sharing the protein major histocompatibility complex class II, DR beta 1 (HLA-DRB1), which is directly involved in the processing and presentation of mycobacterial antigens, crucial to control this infection (64). Noteworthy, the term “Antigen processing and presentation” was commonly represented in both species, although HLA-DRB1 was only significantly expressed in porcine granulomas. Furthermore, the GO term “Selective autophagy” was also identified from DEPs in porcine, which is in connection with the role proposed for neutrophils during TB (55) as well as with the higher infiltrate of neutrophils observed in granulomas from pigs in the present study. Autophagy plays a crucial role in maintaining cellular homeostasis, and dysregulation of this process has been linked to various diseases such as TB, with suppression of this pathway increasing bacterial survival in macrophages as well as a worse prognosis and increased severity of the disease (65).

Regarding the limitations of this study, it should be noted that *in situ* digestion was performed using formalin-fixed and paraffin-embedded tissues in the MALDI-MSI technique process developed in this study. In these tissues, methylene bridges are frequent, which typically affect the efficiency of enzymatic digestion by preventing access to specific cleavage sites of the enzymes. Therefore, some molecules due to their spatial arrangement and/or their relative abundance on the tissue surface may not be accessible to generate peptides from it. Additionally, due to the detection limit of the equipment used, it may not have been possible to detect peptides from some minority proteins that other way could be generated by *in situ* digestion from frozen tissue.

## 5 Conclusions

New high-throughput sequencing techniques, combined with tissue morphology analysis, such as MALDI-MSI, offer a significant advantage for studying the pathogenesis of diseases such as TB. In this study, a proteomic analysis was carried out on cattle and pig tuberculous granulomas identifying some GO-enriched terms shared among both species, such as “Tricarboxylic acid cycle”, “Complement activation, alternative pathway”, and “Regulation of cysteine-type endopeptidase activity involved in apoptotic signaling pathway”, which highlights pathways that are conserved among different species infected by the MTC. Furthermore, species-specific terms identified in the current study, such as “Natural killer cell degranulation” in cattle or those related to platelet and neutrophil recruitment and activation in pigs, provide new insights into host–pathogen interaction in TB in different species and highlight the importance of studying proteomics in understanding this disease.

## Data availability statement

The mass spectrometry proteomics data have been deposited to the ProteomeXchange Consortium (http://proteomecentral.proteomexchange.org) via the PRIDE (66) partner repository with the dataset identifier PXD045377.

## Ethics statement

Ethical review and approval were not required for this animal study since no purposed killing of animals was addressed.

## Author contributions

FL: Formal analysis, Investigation, Visualization, Writing – original draft. JS: Investigation, Writing – review & editing. IR: Investigation, Writing – review & editing. CÁ: Validation, Writing – review & editing. KF: Validation, Writing – review & editing. FP: Validation, Writing – review & editing. LC: Conceptualization, Funding acquisition, Resources, Supervision, Writing – review & editing. EC: Data curation, Formal analysis, Writing – review & editing. IR: Conceptualization, Methodology, Supervision, Writing – review & editing. JG: Conceptualization, Funding acquisition, Methodology, Resources, Supervision, Writing – review & editing.

## Supplementary material

Supplementary Figure 1 Immunohistochemistry for HLA-DR in porcine **(A, B)** and C3b for bovine **(C, D)** in healthy lymph nodes. **(A)** Porcine HLA-DR+ cells. Expression in dendritic-like cells and macrophage-like cells in diffuse lymphoid tissue **(B)**. Detail of HLA-DR+ cells at higher magnification. **(C)** Bovine C3b+ cells. Scattered expression in dentritic-like cells and macrophage-like cells in diffuse lymphoid tissue. **(D)** Detail of C3b+ cells at higher magnification.

Supplementary Figure 2 Immunohistochemistry for MMP9 for bovine and porcine MMP9 in healthy lymph nodes. **(A, C)** Bovine and porcine MMP9+ cells, respectively. Scattered immunolabelled monocyte-like and macrophage-like cells. **(B, D)** Detail of MMP9+ cells at higher magnification in bovine and porcine lymph nodes.
