# Supplementary figures and images for: Proteomic analysis of granulomas from cattle and pigs naturally infected with Mycobacterium tuberculosis complex by MALDI imaging
Source: Front Immunol. 2024 Jul 3;15:1369278. doi: 10.3389/fimmu.2024.1369278 (PMC11252589; doi:10.3389/fimmu.2024.1369278)

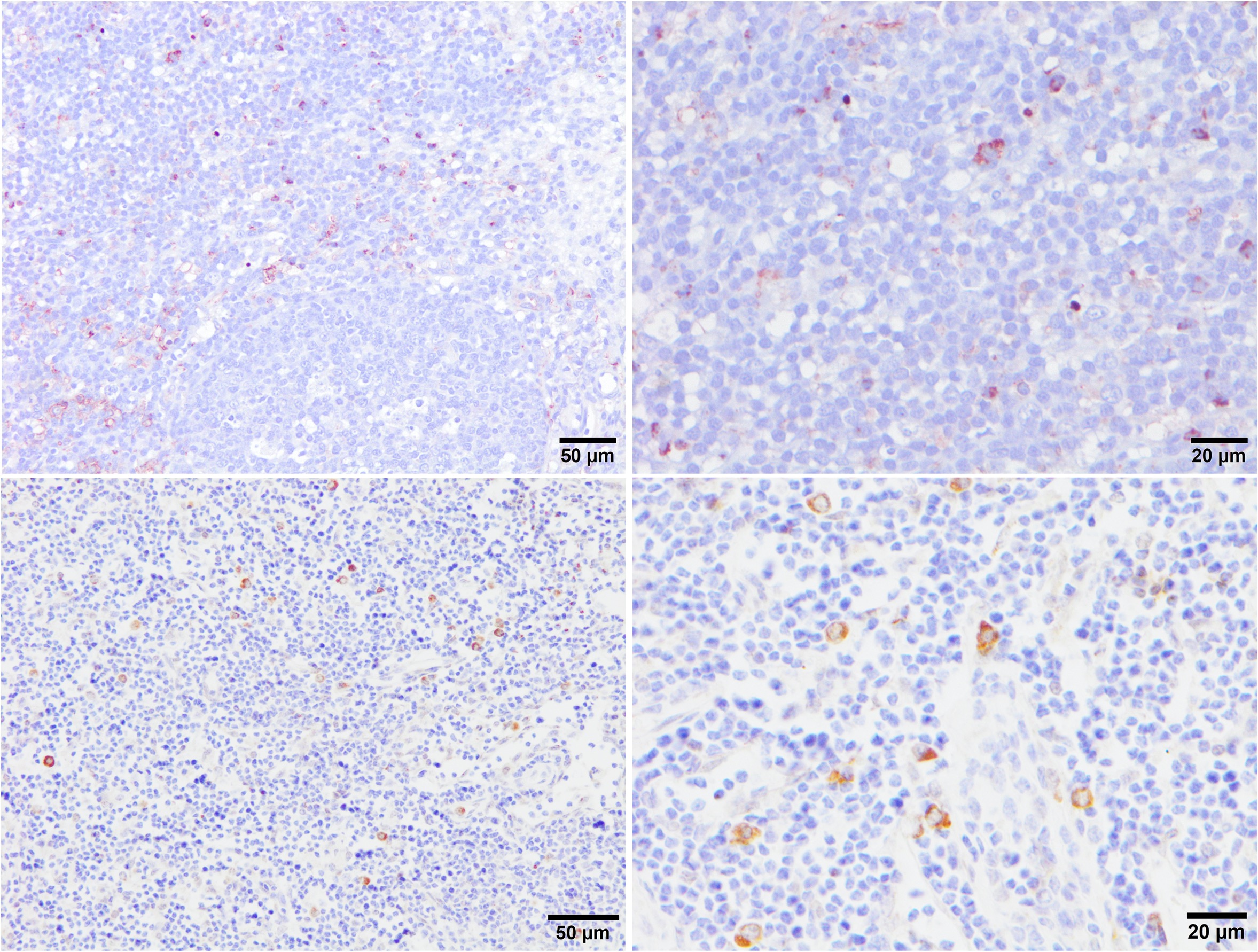

Supplement: Supplementary Figure 1 — Immunohistochemistry for HLA-DR in porcine (A, B) and C3b for bovine (C, D) in healthy lymph nodes. (A) Porcine HLA-DR+ cells. Expression in dendritic-like cells and macrophage-like cells in diffuse lymphoid tissue (B). Detail of HLA-DR+ cells at higher magnification. (C) Bovine C3b+ cells. Scattered expression in dentritic-like cells and macrophage-like cells in diffuse lymphoid tissue. (D) Detail of C3b+ cells at higher magnification. [file Image_1.jpeg]

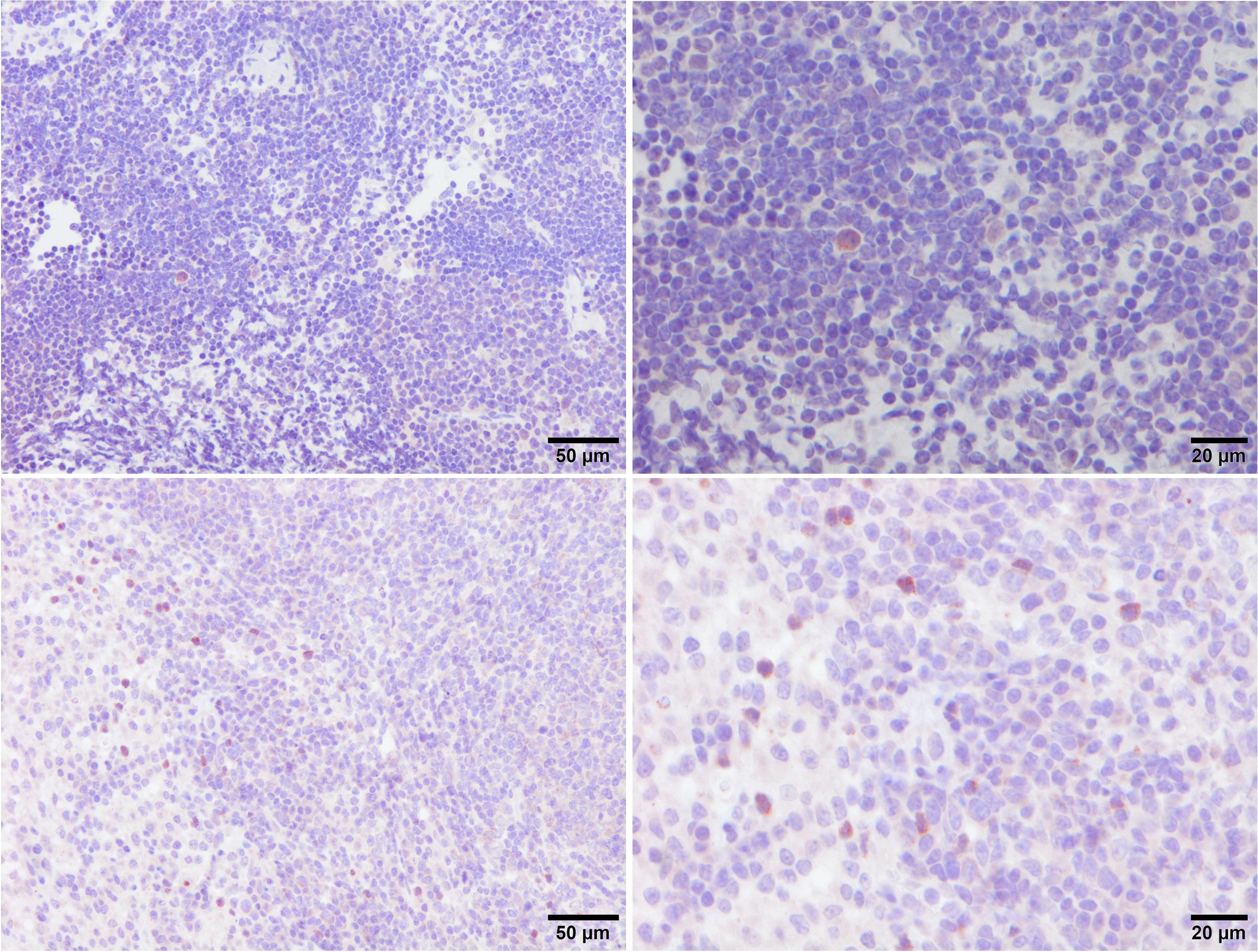

Supplement: Supplementary Figure 2 — Immunohistochemistry for MMP9 for bovine and porcine MMP9 in healthy lymph nodes. (A, C) Bovine and porcine MMP9+ cells, respectively. Scattered immunolabelled monocyte-like and macrophage-like cells. (B, D) Detail of MMP9+ cells at higher magnification in bovine and porcine lymph nodes. [file Image_2.jpeg]
