# Supplementary material for: Proteomic analysis of granulomas from cattle and pigs naturally infected with Mycobacterium tuberculosis complex by MALDI imaging
Source: Front Immunol. 2024 Jul 3;15:1369278. doi: 10.3389/fimmu.2024.1369278 (PMC11252589; doi:10.3389/fimmu.2024.1369278)
Supplement: Supplementary file 3 [file Table_1.docx]

**Table S-1**. Table lists with all terms of Gene Ontology (GO) Biological Processes (BPs), Immune System Processes (ISPs) and Kyoto Encyclopedia of Genes and Genomes (KEGG) and their specific m/z identified in cattle.

| **GO Term** | **No. Proteins** | **% Associated Proteins** | **Associated Proteins Found** |
| --- | --- | --- | --- |
| Citrate cycle (TCA cycle) | 7 | 23.33 | [ACLY, DLD, DLST, IDH1, PCK2, PDHB, SUCLA2] |
| Tricarboxylic acid cycle | 6 | 17.65 | [CFH, DLST, IDH1, NNT, PDHB, SUCLA2] |
| Beta-alanine metabolism | 5 | 16.13 | [ACADS, ACOX1, ALDH2, CNDP2, HADHA] |
| Propanoate metabolism | 5 | 15.63 | [ACADS, ACOX1, DLD, HADHA, SUCLA2] |
| Regulation of complement activation | 4 | 18.18 | [A2M, C3, CFH, PHB1] |
| mRNA cis splicing, via spliceosome | 4 | 16.67 | [DCPS, SNRNP200, SNRPC, SRSF1] |
| Skeletal muscle myosin thick filament assembly | 3 | 75.00 | [MYH11, TTN, TTR] |
| Striated muscle myosin thick filament assembly | 3 | 75.00 | [MYH11, TTN, TTR] |
| Myosin filament assembly | 3 | 60.00 | [MYH11, TTN, TTR] |
| Myosin filament organization | 3 | 50.00 | [MYH11, TTN, TTR] |
| Post-embryonic eye morphogenesis | 3 | 37.50 | [BAX, FBN1, HMGN1] |
| Skeletal myofibril assembly | 3 | 30.00 | [MYH11, TTN, TTR] |
| Oxaloacetate metabolic process | 3 | 27.27 | [ACLY, GOT2, PCK2] |
| Natural killer cell degranulation | 3 | 23.08 | [CORO1A, RAB27A, VAMP7] |
| Nitrogen metabolism | 3 | 17.65 | [CA12, CA2, CPS1] |
| Srp-dependent cotranslational protein targeting to membrane | 3 | 16.67 | [SRP72, SRP9, SRPRB] |
| Regulation of cysteine-type endopeptidase activity involved in apoptotic signalling pathway | 3 | 16.67 | [BAX, FASN, MMP9] |
| Vesicle transport along actin filament | 3 | 15.79 | [ACTN4, MYO1D, MYO5C] |
| Complement activation, alternative pathway | 3 | 15.00 | [C3, C9, CFH] |
| Nucleotide-sugar biosynthetic process | 3 | 15.00 | [GFPT1, GMPPB, NANS] |
| Regulation of early endosome to late endosome transport | 3 | 15.00 | [DNAJC13, MAP2K1, RAB21] |
| 2-oxoglutarate metabolic process | 3 | 1.00 | [DLST, GOT2, IDH1] |

***No.: number.***
