# Supplementary material for: Proteomic analysis of granulomas from cattle and pigs naturally infected with Mycobacterium tuberculosis complex by MALDI imaging
Source: Front Immunol. 2024 Jul 3;15:1369278. doi: 10.3389/fimmu.2024.1369278 (PMC11252589; doi:10.3389/fimmu.2024.1369278)
Supplement: Supplementary file 4 [file Table_2.docx]

**Table S-2**. Table lists with all terms of Gene Ontology (GO) Biological Processes (BPs), Immune System Processes (ISPs) and Kyoto Encyclopedia of Genes and Genomes (KEGG) and their specific m/z identified in pigs.

| **GO Term** | **No. Proteins** | **% Associated Proteins** | **Associated Proteins Found** |
| --- | --- | --- | --- |
| Platelet aggregation | 14 | 18.42 | [ACTB, ACTN1, CEACAM1, CSRP1, CTSG, FGA, FGB, FGG, FN1, HBB, HSPB1, MYH9, PTPN6, TLN1] |
| Glycolysis/gluconeogenesis | 14 | 20.90 | [ACSS1, ALDH2, ENO3, GAPDH, GPI, HK2, LDHA, LDHB, PCK2, PDHB, PFKL, PFKP, PGK1, TPI1] |
| DNA replication-dependent chromatin assembly | 11 | 34.38 | [H3C1, H3C10, H3C11, H3C12, H3C2, H3C3, H3C4, H3C6, H3C7, H3C8, H4C1] |
| Myofibril assembly | 11 | 15.71 | [CFL2, CSRP1, CSRP3, FLNC, ITGB1, MYH11, MYH7, MYOZ1, PGM5, TPM1, TTN] |
| DNA replication-dependent chromatin organization | 11 | 34.38 | [H3C1, H3C10, H3C11, H3C12, H3C2, H3C3, H3C4, H3C6, H3C7, H3C8, H4C1] |
| Sarcomere organization | 9 | 20.00 | [CFL2, CSRP1, CSRP3, FLNC, ITGB1, MYH7, MYOZ1, TPM1, TTN] |
| Tricarboxylic acid cycle | 7 | 20.59 | [ACO1, CFH, CS, DLST, IDH2, NNT, PDHB] |
| Canonical glycolysis | 6 | 25.00 | [ENO3, HK2, PFKL, PFKP, PGK1, TPI1] |
| Glucose catabolic process to pyruvate | 6 | 25.00 | [ENO3, HK2, PFKL, PFKP, PGK1, TPI1] |
| Glycolytic process through glucose-6-phosphate | 6 | 23.08 | [ENO3, HK2, PFKL, PFKP, PGK1, TPI1] |
| Glycolytic process through fructose-6-phosphate | 6 | 22.22 | [ENO3, HK2, PFKL, PFKP, PGK1, TPI1] |
| Citrate cycle (TCA cycle) | 6 | 20.00 | [ACO1, CS, DLST, IDH2, PCK2, PDHB] |
| Glucose catabolic process | 6 | 18.75 | [ENO3, HK2, PFKL, PFKP, PGK1, TPI1] |
| Glutamine metabolic process | 6 | 1.93 | [ADSL, CTPS1, GFPT1, GLS, MECP2, PHGDH] |
| Pentose phosphate pathway | 5 | 16.67 | [G6PD, GPI, PFKL, PFKP, TALDO1] |
| Glutamine family amino acid biosynthetic process | 4 | 21.05 | [ADSL, ALDH18A1, GLS, OTC] |
| Regulation of dopamine metabolic process | 4 | 20.00 | [ALDH2, ITGAM, MAOB, VPS35] |
| Regulation of catecholamine metabolic process | 4 | 20.00 | [ALDH2, ITGAM, MAOB, VPS35] |
| Regulation of superoxide anion generation | 4 | 16.00 | [ACP5, CSRP1, ITGAM, SOD1] |
| Regulation of cardiac muscle contraction by calcium ion signaling | 4 | 15.38 | [ATP2A2, CALM1, DMD, GSTM2] |
| Basolateral protein secretion | 3 | 75.00 | [AP1B1, AP1G1, AP1S1] |
| Proteasome-activating activity | 3 | 50.00 | [PSMC2, PSMC5, PSMC6] |
| Regulation of collagen fibril organization | 3 | 42.86 | [COLGALT1, EMILIN1, TNXB] |
| Positive regulation of establishment of protein localization to telomere | 3 | 30.00 | [CCT2, CCT7, CCT8] |
| Very-low-density lipoprotein particle assembly | 3 | 27.27 | [APOB, DGAT1, LPCAT3] |
| Regulation of establishment of protein localization to telomere | 3 | 27.27 | [CCT2, CCT7, CCT8] |
| Regulation of protein localization to Cajal body | 3 | 27.27 | [CCT2, CCT7, CCT8] |
| Positive regulation of protein localization to Cajal body | 3 | 27.27 | [CCT2, CCT7, CCT8] |
| Elastic fiber assembly | 3 | 25.00 | [EMILIN1, MYH11, TNXB] |
| Regulation of establishment of protein localization to chromosome | 3 | 25.00 | [CCT2, CCT7, CCT8] |
| Neutrophil-mediated killing of bacterium | 3 | 25.00 | [AZU1, CTSG, F2] |
| Protein localization to nuclear body | 3 | 25,00 | [CCT2, CCT7, CCT8] |
| Positive regulation of protein localization to chromosome, telomeric region | 3 | 25.00 | [CCT2, CCT7, CCT8] |
| Protein localization to Cajal body | 3 | 25.00 | [CCT2, CCT7, CCT8] |
| Spliceosomal tri-snrnp complex assembly | 3 | 23.08 | [LSM2, PRPF8, SART3] |
| ATP transmembrane transporter activity | 3 | 23.08 | [SLC25A24, SLC25A4, SLC25A5] |
| Ornithine metabolic process | 3 | 23.08 | [ADSL, ALDH18A1, OTC] |
| Negative regulation of cell morphogenesis involved in differentiation | 3 | 21.43 | [CORO1C, FBLN1, GBP1] |
| Neutrophil-mediated killing of symbiont cell | 3 | 21.43 | [AZU1, CTSG, F2] |
| Negative regulation of substrate adhesion-dependent cell spreading | 3 | 21.43 | [CORO1C, FBLN1, GBP1] |
| Regulation of protein localization to chromosome, telomeric region | 3 | 21.43 | [CCT2, CCT7, CCT8] |
| Protein localization to nucleoplasm | 3 | 21.43 | [CCT2, CCT7, CCT8] |
| Phospholipase inhibitor activity | 3 | 20.00 | [ANXA1, ANXA4, ANXA5] |
| Positive regulation of heterotypic cell-cell adhesion | 3 | 20.00 | [FGA, FGB, FGG] |
| Regulation of skeletal muscle contraction | 3 | 18.75 | [DMD, GSTM2, MYH7] |
| Positive regulation of telomerase RNA localization to Cajal body | 3 | 18.75 | [CCT2, CCT7, CCT8] |
| Phenylalanine metabolism | 3 | 18.75 | [AOC3, GOT2, MAOB] |
| Establishment of protein localization to telomere | 3 | 17.65 | [CCT2, CCT7, CCT8] |
| ATP transport | 3 | 16.67 | [SLC25A24, SLC25A4, SLC25A5] |
| Regulation of cysteine-type endopeptidase activity involved in apoptotic signaling pathway | 3 | 16.67 | [GSN, HTRA2, MMP9] |
| Adenine nucleotide transmembrane transporter activity | 3 | 15.79 | [SLC25A24, SLC25A4, SLC25A5] |
| Purine ribonucleotide transmembrane transporter activity | 3 | 15.79 | [SLC25A24, SLC25A4, SLC25A5] |
| Vesicle transport along actin filament | 3 | 15.79 | [MYO1D, MYO5C, MYO6] |
| Negative regulation of natural killer cell mediated cytotoxicity | 3 | 15,79 | [CEACAM1, HLA-A, HLA-B] |
| Regulation of telomerase RNA localization to Cajal body | 3 | 15.79 | [CCT2, CCT7, CCT8] |
| Negative regulation of natural killer cell mediated immunity | 3 | 15.00 | [CEACAM1, HLA-A, HLA-B] |
| 2-oxoglutarate metabolic process | 3 | 15.00 | [DLST, GOT2, IDH2] |
| Complement activation, alternative pathway | 3 | 15.00 | [C8G, C9, CFH] |
| Peptidyl-cysteine S-nitrosylation | 3 | 15.00 | [DMD, GAPDH, S100A8] |
| Platelet formation | 3 | 15.00 | [ACTN1, MYH9, PTPN6] |
| Melanosome assembly | 3 | 15,00 | [AP1B1, AP1G1, AP1S1] |

***No.: number.***
